# The Intention of Primary Health Nurses to Participate in Internet Plus Nursing Service: Cross-Sectional Survey

**DOI:** 10.2196/72846

**Published:** 2025-10-01

**Authors:** Bin Xu, Suyuan Li, Keyu Chen, Lei Ding, Longxiu Liu

**Affiliations:** 1 Neurosurgical Intensive Care Unit, Nanjing Medical University First Affiliated Hospital, Nanjing, Jiangsu Province, China; 2 School of Nursing, Nanjing Medical University, Nanjing, Jiangsu Province, China; 3 Urology Department, Jiangsu Province Hospital and Nanjing Medical University First Affiliated Hospital, Nanjing, Jiangsu Province, China; 4 Coronary Care Unit, Jiangsu Province Hospital and Nanjing Medical University First Affiliated Hospital, Nanjing, Jiangsu Province, China; 5 Nursing Department, Changzhou Maternal and Child Health Care Hospital, No. 16 Dingxiang Road, Zhonglou District, Changzhou, Jiangsu Province, 213023, China, 8618761163935; 6 Nursing Department, Jiangsu Province Hospital and Nanjing Medical University First Affiliated Hospital, 300 Guangzhou Road, Nanjing, Nanjing, Jiangsu Province, China, 86 13921431834

**Keywords:** cross-sectional studies, home health care, internet-based nursing, participation intention, primary health nurse

## Abstract

**Background:**

“Internet Plus Nursing Service” (IPNS) offers innovative solutions for China’s growing home health care demands. Understanding primary care nurses’ participation intentions is crucial for service optimization.

**Objective:**

This study evaluates primary health nurses’ intention to participate in IPNS—a technology-mediated home care model combining mobile health platforms with in-person visits—and examines how digital readiness, safety perceptions, and organizational factors influence participation decisions, to guide policy optimization for scalable digital home health care delivery.

**Methods:**

A cross-sectional survey was conducted in Jiangsu Province, China (December 2023-December 2024) using the validated Participation Intention of Nurses on IPNS Scale. Convenience sampling enrolled 3952 nurses from 13 prefecture-level cities in Jiangsu—the second-tier administrative divisions in China that typically encompass both urban and rural areas, each with independent health care systems governed by municipal health authorities. Statistical analyses included *t* tests and ANOVA with SPSS 22.

**Results:**

A total of 3952 surveys were completed. The participation intention scale yielded a mean (SD) total score of 66.13 (7.89) across respondents. Subscale analysis revealed mean (SD) scores of 18.57 (2.68) for participation attitude, 18.87 (2.49) for subjective norms, and 25.67 (3.48) for perceived behavioral control. Significant demographic predictors of participation intention were identified through statistical analysis. Male nurses demonstrated stronger intention (*t_72.974_*=−23.139, *P*<.0001), as did those over 30 years old (*F_39,51_*=27.215, *P*<.0001) and bachelor’s degree holders (*t_2185.018_*=−4.994, *P*<.0001). Workplace characteristics also showed significant associations, with nursing management department staff (*F_39,51_*=45.877, *P*<.0001) and those with less organizational workloads (*F_39,51_*=9.829, *P*<.0001) displaying greater intention. Professional factors including higher positional rank (*F_39,51_*=37.32, *P*<.0001), more advanced titles (*F_39,51_*=30.176, *P*<.0001), and over 11 years of experience (*F_39,51_*=5.242, *P*=.001) predicted stronger participation intent. Finally, nurses earning 5000‐10,000 RMB (a currency exchange rate of RMB 1=US $0.71 is applicable) monthly showed significantly higher intention scores (*F_39,51_*=16.141, *P*<.0001).

**Conclusions:**

Policymakers should prioritize 3 interventions: (1) develop IPNS-specific safety protocols and legal safeguards, (2) optimize workload allocation through intelligent scheduling systems, and (3) establish tiered incentive mechanisms targeting middle-income nurses and experienced practitioners.

## Introduction

“Internet Plus Nursing Service” (IPNS) has gradually gained attention in China as a new model for extending nursing services. This model combines traditional health care with digital technology, allowing registered nurses to use online platforms, such as mobile apps or systems affiliated with hospitals, to offer integrated care to patients who have been discharged or those with limited mobility. Through this service, nurses visit patients’ homes to provide a wide range of care, including wound management and chronic disease treatment. The entire process, from scheduling appointments to tracking services in real-time and documenting visits, is managed digitally. This shift enables nursing services to move from conventional medical institutions to community and home environments, marking a stark departure from traditional home nursing methods that often depend on phone calls or require patients to visit health care centers for service arrangements. In recent times, there has been a notable increase in demand for this “online application, doorstep service” model in China. To qualify for IPNS, nurses must have at least 5 years of clinical experience and hold a senior professional title, and they must be verifiable through the national nurse registration systems [1].

With China’s aging population and changing disease patterns, there is an increasing demand for home health care. By 2020, over 264 million people aged 60 years and above lived in China, accounting for 18.7% of the total population, an increase of 5.4% since 2010 [2]. The World Health Organization predicts that by 2050, China will have the largest elderly population in the world [3]. Additionally, around 260 million people in China are living with chronic diseases, and this number continues to rise [4]. The country also has 85 million disabled people, more than half of whom require long-term care at home [5]. Although China has over 4.7 million registered nurses, with 1.15 million working in primary health institutions, the nurse-to-population ratio is only 3.56 per 1000 people. This indicates a significant gap in the availability of nursing services.

Home-based health care has seen extensive implementation in high-income countries like the United Kingdom and the United States, largely driven by rapid advancements in information technology. This transformation has led to the emergence of IPNS, which plays a crucial role in enhancing the quality and efficiency of care. The main goals of IPNS include facilitating patient recovery, minimizing waste of medical resources, and reducing health care costs [6,7]. The evolution of health care policy in the United Kingdom, from the establishment of the National Health Service to its recent digital transformation, showcases a continuous adaptation to the changing public health landscape [8]. In recent years, the UK government has prioritized digital health technologies to tackle challenges such as an aging population and the increasing prevalence of chronic diseases, aiming to improve service efficiency and patient experiences [8,9]. In both the United States and the United Kingdom, IPNS is primarily delivered through mobile health and telenursing. Mobile health provides health information and services via mobile devices, significantly enhancing patients’ ability to manage their own health, especially in the context of chronic disease management [10]. Patients can utilize dedicated apps to record and monitor their health data, which supports more effective self-care [11]. Additionally, wearable devices and environmental sensors enable health care providers to remotely monitor patients’ physiological parameters in real time. The data analytics generated from these technologies assist clinicians in creating personalized treatment plans, ultimately leading to improved therapeutic outcomes [12].

In response to the challenges posed by an aging population and a shortage of nursing personnel, IPNS has been piloted across China with support from “Internet Plus” technology and national policies. However, it is still in the early stages of development. The IPNS model, also known as “Sharing Nurse,” was first introduced in Shandong Province in 2016 and has since expanded to other regions, including Beijing, Shanghai, and Guangzhou [13]. In 2018, the State Council issued the “Opinions on Promoting the Development of ‘Internet Plus’ Medical and Health Care,” emphasizing that “Internet Plus” health care is a critical component of the “Healthy China” initiative [14]. Subsequently, the National Health Commission issued the “Pilot Work Plan for ‘Internet Plus Nursing Service’” to increase the supply of nursing services through IPNS and meet patients’ diverse health care needs more effectively [15]. IPNS is part of the broader movement toward integrating “Internet Plus” technology into health care, supporting the development of both “Healthy China” and “Digital China.”

IPNS allows patients to receive care at home without visiting health care institutions, addressing the challenge of the “last mile” in health care delivery. Nurses are essential to the success of IPNS, and their willingness to participate will directly influence the implementation and expansion of the service. Previous research has explored nurses’ perceptions of IPNS, but little is known about primary health care nurses’ intentions to participate. This study seeks to fill this gap by assessing the participation intentions of nurses in primary health institutions, aiming to provide recommendations for policy development and strategies to recruit more nurses into IPNS.

## Methods

### Design and Setting of the Study

This cross-sectional survey was conducted in Jiangsu Province, China, between December 2023 and December 2024. Registered nurses from 13 prefecture-level cities in Jiangsu were recruited using stratified convenience sampling, encompassing both urban and rural administrative divisions. Recruitment ensured representation from different types of primary health care institutions. The inclusion criteria for participation were (1) being a registered nurse working at a primary health care institution, (2) having at least 1 year of clinical experience, and (3) agreeing to participate in the survey. The exclusion criteria were (1) being away from nursing-related duties for more than 12 months and (2) experiencing significant personal or family events within the past 6 months, such as bereavement or divorce.

Of the 4016 nurses who initially completed the survey, 64 were excluded from analysis: 19 had missing responses exceeding 20%, 17 met the exclusion criteria, and 28 provided straight-line responses. The final sample consisted of 3952 nurses.

### Survey Questionnaire

The data were collected using a self-designed questionnaire that included sociodemographic information and the “Participation Intention of Nurses on Internet Plus Nursing Service Scale,” developed by Su et al [16]. Guided by the Theory of Planned Behavior by Young et al [17], we conceptualized IPNS participation intention as driven by 3 latent constructs: attitude—personal evaluation of participation outcomes, subjective norms—perceived social expectations, and perceived control—self-efficacy assessments. This theoretical orientation informed our scale development and item formulation. The questionnaire was administered online via a platform.

### Sociodemographic Information

Sociodemographic variables included age, gender, educational background, department, organizational workload, position, professional title, years of experience, personal income, marital status, and number of children.

### Participation Intention of Nurses on Internet Plus Nursing Service Scale

The “Participation Intention of Nurses on Internet Plus Nursing Service Scale” was specifically designed to measure nurses’ intentions to participate in IPNS. The scale comprises 3 subscales: participation attitude (5 items), subjective norms (5 items), and perceived behavioral control (7 items). The participation attitude subscale includes items related to the development concept (A1-A2), values (A3-A4), and economic outlook (A5). The subjective norms subscale addresses work-related (B1-B3) and family-related (B4-B5) influences. The perceived behavioral control subscale evaluates self-perception (C1-C2), environmental perception (C3-C5), and technological perception (C6-C7).

Each item is rated on a 5-point Likert scale, with responses ranging from 1 (strongly disagree) to 5 (strongly agree). The total score on the scale ranges from 7 to 85, with reverse scoring applied to items 10, 12, and 13 (where original scores of 1=5, 2=4, 3=3, 4=2, and 5=1 were transformed before summation). Higher scores indicate a stronger intention to participate in IPNS. The internal consistency of the scale was confirmed with a Cronbach α of .871 in this study, indicating good reliability.

### Data Collection and Analysis

The survey was distributed online from December 2023 to December 2024. Categorical data were summarized using frequencies and percentages, while normally distributed continuous data were reported as mean (standard deviation). To assess differences in participation intention based on demographic characteristics, independent *t* tests and one-way ANOVA were employed. Post hoc testing using the least significant difference method was applied where applicable. A *P* value of less than .05 was considered statistically significant. Data analysis was performed using SPSS version 22 (IBM Corp.).

### Ethical Considerations

For privacy protection, in compliance with relevant regulations, participants were informed of the scope and purpose of personal privacy information collection, with only information essential for the study collected after obtaining their consent, and additional consent from legal guardians secured for those from special groups. Regarding data confidentiality, participants' personal identification information was separated from core research data and anonymized with codes; the data were stored on encrypted servers, transmitted via encrypted channels, accessible only to authorized researchers, and archived or destroyed in accordance with regulations after the study. For participant compensation, reasonable compensation (RMB 1-2 [US $ 0.71~1.42]) was provided based on participants' time and effort invested, with reimbursement available for additional expenses incurred, and the compensation was independent of study results with a compliant funding source. Ethically, the study was approved by the Ethics Committee of Nanjing Medical University First Affiliated Hospital (approval number: 2023-SRFA-247).

## Results

### Sociodemographic Information and Work-Related Characteristics of Primary Health Care Nurses

A total of 3952 primary health care nurses participated in the study. Of these, 68 (1.8%) were men and 3884 (98.2%) were women. The ages of the participants ranged from 21 to 56 years, with a mean (SD) age of 32.35 (8.57) years. The average (SD) years of clinical nursing experience was 11.6 (6.9) years. Regarding educational background, 1128/3952 (31.1%) held an associate’s degree, while 2724/3952 (68.9%) had a bachelor’s degree.

The distribution of departments in which the nurses worked was as follows: 1452/3952 (36.7%) worked in internal medicine, 1248/3952 (31.6%) in surgery, 564/3952 (14.3%) in nursing management, 408/3952 (10.3%) in gynecology and pediatrics, and 208/3952 (5.3%) in other departments.

Regarding the perception of organizational workload, 84/3952 (2.1%) nurses felt their workload was excessive, 2194/3952 (55.5%) considered their workload heavy, and 1674/3952 (42.4%) felt their workload was normal.

With respect to professional titles, 2656/3952 (67.2%) were general nurses, 768/3952 (19.4%) were specialist nurses, 388/3952 (9.8%) were head nurses, and 140/3952 (3.5%) were directors of nursing. Regarding seniority, 2164/3952 (54.8%) were primary nurses, 800/3952 (20.2%) were intermediate nurses, and 988/3952 (25%) were senior nurses.

In terms of income, 2460/3952 (54.7%) nurses earned less than 5000 RMB per month, 1516/3952 (38.4%) earned between 5000 and 10,000 RMB per month, and 276/3952 (6.9%) earned more than 10,000 RMB per month; a currency exchange rate of RMB 1=US $0.71 is applicable. Details can be found in Table 1.

**Table 1.** Participation intention to Internet Plus Nursing Service (IPNS) of primary health care nurses (N=3952).

		Participation intention scores				
Items	Participants, n (%)	Mean (SD)	*t* test *(df)*	*F* test *(df)*	*P* value	Post hoc
Gender			–23.139 (72.974)	N/A^a^	<.001	N/A
Female	3884 (98.3)	64.23 (8.33)				
Male	68 (1.8)	79.35 5.27)				
Age (years)			N/A	27.215 (39, 51)	<.001	B>A C>A
A <30	1676 (42.4)	63.34 (8.75)				
B 30‐40	972 (24.6)	65.30 (8.69)				
C >40	1304 (33)	65.38 (7.88)				
Education background			–4.994 (2185.018)	N/A	<.001	N/A
Associate’s degree	1228 (31.1)	63.46 (9.01)				
Bachelor’s degree	2724 (68.9)	65.96 (8.24)				
Departments			N/A	45.877 (39, 51)	<.001	A>B-E
A Nursing management	564 (14.3)	67.52 (8.32)				
B Internal medicine	1452 (36.1)	62.74 (5.18)				
C Surgery	1248 (31.6)	63.40 (9.08)				
D Gynecology and pediatrics	408 (10.3)	63.64 (8.73)				
E Others	208 (5.3)	63.57 (7.66)				
Organization workload			N/A	9.829 (39, 51)	<.001	C>B>A
A Excessive	84 (2.1)	60.58 (10.85)				
B Heavy	2194 (55.5)	64.44 (8.89)				
C Normal	1674 (42.4)	66.77 (7.81)				
Position				37.32 (39, 51)	<.001	D>C>A-B
A Nurse	2656 (67.2)	63.73 (8.28)				
B Specialist nurses	768 (19.4)	62.83 (4.80)				
C Head nurse	388 (9.8)	65.66 (10.14)				
D Director of nursing	140 (3.5)	68.05 (6.12)				
Professional titles			N/A	30.176 (39, 51)	<.001	C>B>A
A Primary	2164 (54.8)	63.90 (8.47)				
B Intermediate	800 (20.2)	64.86 (7.96)				
C Senior	988 (25.0)	66.30 (8.78)				
Years of working			N/A	5.242 (39, 51)	.001	C>A-B D>A-B
A 1-5	1176 (29.8)	64.02 (9.19)				
B 5-10	776 (19.6)	63.89 (7.75)				
C 11-20	756 (19.1)	64.78 (8.70)				
D >20	1244 (31.5)	65.14 (8.13)				
Personal income (RMB^b^^c^ per month）			N/A	16.141 (39, 51)	<.001	B>A B>C
A 1-5000	2160 (54.7)	63.80 (8.42)				
B 5000-10,000	1516 (38.4)	65.27 (8.63)				
C >10,000	276 (6.9)	63.65 (8.09)				
Total	3952 (100)	66.13 (7.89)				

a N/A: not applicable.

b RMB: Renminbi (Chinese Yuan), the official currency of China.

c A currency exchange rate of RMB 1=US $0.71 is applicable.

### Intention to Participate in IPNS

The mean (SD) total score on the Participation Intention Scale was 66.13 (7.89). The mean (SD) score for the participation attitude subscale was 18.57 (2.68). Major primary health care nurses viewed IPNS as a new opportunity for self-actualization (3256/3952, 82.4%), perceiving it as a way to enhance their professional image (3136/3952, 79.4%), increase the value of nursing (3272/3952, 82.8%), and improve their income (3120/3952, 78.9%). However, more than half of the nurses expressed concerns, believing that the risks of IPNS outweighed its benefits.

The mean (SD) score for the subjective norms subscale was 18.87 (2.49). Most nurses reported they would participate in IPNS based on the recommendations of hospital leaders (3344/3952, 84.6%), colleagues (3200/3952, 81.0%), experts (3260/3952, 82.5%), or relatives (2858/3952, 72.3%). However, 1708/3952 (43.2%) participants noted that IPNS might require significant amounts of their time, which could be a barrier to their participation.

The mean (SD) score for the perceived behavioral control subscale was 25.67 (3.48). A large proportion of nurses reported feeling satisfied with serving more patients (2844/3952, 71.9%) and had a positive perception of IPNS policies (2836/3952, 71.8%) and associated risks (2776/3952, 70.2%). Additionally, 2604/3952 (65.9%) nurses expressed confidence in the technological aspects of IPNS. However, 2092/3952 (52.9%) nurses expressed concerns that a change in their physical location might negatively impact the quality of care they could provide. The details are presented in Table 2.

**Table 2.** Participation intention to Internet Plus Nursing Service (IPNS) of primary health care nurses (N=3952).

Dimensions/item	Strongly disagree, n (%)	Partially disagree, n (%)	Neither agree nor disagree, n (%)	Partially agree, n (%)	Strongly agree, n(%)
A. Participation attitude					
A1. I think “Internet+ nursing service” is a new thing and a path of self-realization	28 (0.7)	68 (1.7)	668 (16.9)	1824 (46.2)	1432 (36.2)
A2. I think participating in the “Internet+nursing service” can shape my personal image and expand my influence	28 (0.7)	80 (2.0)	708 (17.9)	1692 (42.8)	1444 (36.5)
A3. I think participating in “Internet+nursing service” has more advantages than disadvantages and can enhance the professional value of nursing	14 (0.4)	20 (0.5)	660 (16.7)	1808 (45.7)	1464 (37.0)
A4. I think there are more risks than the benefits of participating in “Internet+care services”	72 (1.8)	460 (11.6)	1392 (35.2)	1264 (32.0)	764 (19.3)
A5. I think I can participate in the “Internet+nursing services” to get more benefits	58 (1.5)	160 (4.0)	672 (17.0)	1768 (44.7)	1352 (34.2)
B. Subjective norm					
B1. I will participate in the “Internet+Nursing Service” due to the support and guidance of my hospital leaders	28 (0.7)	53 (1.3)	580 (14.7)	1952 (49.4)	1392 (35.2)
B2. I will participate in the “Internet+nursing Service” for the recommendation of my colleagues in my hospital	28 (0.7)	80 (2.0)	644 (16.3)	1860 (47.1)	1340 (33.9)
B3. I will participate in “Internet+nursing Services” due to the views of experts in related fields	37 (0.9)	24 (0.6)	668 (16.9)	1908 (48.3)	1352 (34.2)
B4. I will participate in the “Internet+nursing Service” due to the advice of my relatives and friends	45 (1.1)	156 (3.9)	908 (23.0)	1712 (43.3)	1176 (29.8)
B5. I am worried that participating in “Internet+nursing services” will take up a lot of my family life time and increase my burden	168 (4.3)	624 (15.8)	1452 (36.7)	1016 (25.7)	692 (17.5)
C. Perceived behavioral control					
C1. I think participating in the “Internet+nursing service” can enable me to serve more patients in need and make me feel happy	14 (0.4)	64 (1.6)	636 (16.1)	1792 (45.3)	1460 (36.9)
C2. I am confident that I can provide good services for patients independently through the “Internet+nursing service”	32 (0.8)	92 (2.3)	1016 (25.7)	1812 (45.9)	1032 (26.1)
C3. I understand the relevant policies of government departments on carrying out “Internet+nursing service,” and I will consider participating in this service	21 (0.5)	36 (0.9)	1080 (27.3)	1756 (44.4)	1080 (27.3)
C4. I think the change of physical location when participating in “Internet+nursing services” will affect the quality of nursing services provided	28 (0.7)	468 (11.8)	1364 (34.5)	1336 (33.8)	756 (19.1)
C5. I understand the risks I may encounter in participating in “Internet+nursing services”	16 (0.4)	128 (3.2)	1048 (26.5)	1784 (45.1)	992 (25.1)
C6. I can get the training on how to participate in the basic technology of “Internet+nursing service”	23 (0.6)	60 (1.5)	1288 (32.6)	1560 (39.5)	1044 (26.4)
C7. I think it is easy to master the registration, certification, audit, login, and other links that you must pass before participating in the “Internet+nursing service”	16 (0.4)	36 (0.9)	920 (23.3)	1832 (46.4)	1148 (29.0)

### Variations in Participation Intentions Based on Demographic Characteristics

Significant differences in participation intentions were observed based on several demographic factors. Male nurses (*t_72.974_*=−23.139,, *P*<.0001), nurses aged over 30 years (*F*_39,51_=27.215, *P*<.0001), those with a bachelor’s degree (*t_2185.018_*=-4.994, *P*<.0001), those working in departments with nursing management (*F*_39,51_=45.877, *P*<.0001), those with a lighter organizational workload (*F*_39,51_=9.829, *P*<.0001), those holding higher positions (*F_39,51_*=37.32, *P*<.0001) or professional titles (*F_39,51_*=30.176, *P*<.0001), those with over 11 years of experience (*F_39,51_*=5.242, *P*=.001), and those with a personal income between 5000 and 10,000 RMB per month (*F_39,51_*=16.141, *P*<.0001) were more likely to express a positive intention to participate in IPNS. Similar results are found in the subscales (Tables1 and 3^5^).

**Table 3.** Participation attitude to Internet Plus Nursing Service (IPNS) of primary health care nurses (N=3952).

Items	Participants, n (%)	Participation attitude scores				
		Mean (SD)	*t* test *(df)*	*F* test *(df)*	*P* value	Post hoc
Gender			–19.831 (72.645)	N/A^a^	<.001	N/A
Female	3884 (98.3)	18.95 (2.75)				
Male	68 (1.8)	23.35 (1.79)				
Age (years)			N/A	57.442 (39, 51)	<.001	B>A C>A
A <30	1676 (42.4)	18.49 (2.76)				
B 30‐40	972 (24.6)	19.27 (3.00)				
C >40	1304 (33)	19.54 (2.57)				
Education background			–5.59 (2303.484)	N/A	<.001	N/A
Associate’s degree	1228 (31.1)	18.66 (2.85)				
Bachelor’s degree	2724 (68.9)	19.19 (2.76)				
Departments			N/A	37.083 (39, 51)	<.001	A>B-E E>B-D
A Nursing management	564 (14.3)	20.04 (2.46)				
B Internal medicine	1452 (36.1)	18.69 (3.07)				
C Surgery	1248 (31.6)	18.45 (3.03)				
D Gynecology and pediatrics	408 (10.3)	18.92 (2.05)				
E Others	208 (5.3)	19.07 (2.33)				
Organization workload			N/A	11.296 (39, 51)	<.001	C>B>A
A Excessive	84 (2.1)	17.6 (3.45)				
B Heavy	2194 (55.5)	19.06 (2.94)				
C Normal	1674 (42.4)	18.05 (2.54)				
Position			N/A	53.939 (39, 51)	<.001	D>C>A-B
A Nurse	2656 (67.2)	18.68 (2.68)				
B Specialist nurses	768 (19.4)	19.00 (1.70)				
C Head nurse	388 (9.8)	19.57 (3.47)				
D Director of nursing	140 (3.5)	20.34 (1.68)				
Professional titles			N/A	44.857 (39, 51)	<.001	C>B>A B>A
A Primary	2164 (54.8)	18.68 (2.75)				
B Intermediate	800 (20.2)	19.19 (2.53)				
C Senior	988 (25.0)	19.66 (2.98)				
Years of working			N/A	16.208 (39, 51)	<.001	C>A-B D>A-B
A 1-5	1176 (29.8)	18.74 (2.84)				
B 5-10	776 (19.6)	18.74 (2.61)				
C 11-20	756 (19.1)	19.09 (2.95)				
D >20	1244 (31.5)	19.44 (2.73)				
Personal income (RMB^b^^c^ per month)			N/A	20.218 (39, 51)	<.001	B>A B>C
A 1-5000	2160 (54.7)	18.77 (2.71)				
B 5000-10,000	1516 (38.4)	19.30 (2.92)				
C >10,000	276 (6.9)	18.49 (2.58)				
Total	3952 (100)	18.57 (2.68)				

a N/A: not applicable.

b RMB: Renminbi (Chinese Yuan), the official currency of China.

c A currency exchange rate of RMB 1=US $0.71 is applicable.

**Table 4.** Subjective norms to Internet Plus Nursing Service (IPNS) of primary health care nurses (N=3952).

	Participants, n (%)	Subjective norm				
Items		Mean (SD)	*t* test *(df)*	*F* test *(df)*	*P* value	Post hoc
Gender			–19.573 (72.614)	N/A^a^	<.001	N/A
Female	3884 (98.2)	19.01 (2.75)				
Male	68 (1.8)	23.35 (1.79)				
Age (years)			N/A	50.727 (39, 51)	<.001	B>A C>A
A <30	1676 (42.4)	18.58 (2.64)				
B 30‐40	972 (24.6)	19.29 (3.02)				
C >40	1304 (33)	19.57 (2.69)				
Education background			–7.48 (2347.998)	N/A	<.001	N/A
Associate’s degree	1228 (31.1)	18.59 (2.79)				
Bachelor’s degree	2724 (68.9)	19.31 (2.76)				
Departments			N/A	34.444 (39, 51)	<.001	A>B-E
A Nursing management	564 (14.3)	19.97 (3.17)				
B Internal medicine	1452 (36.1)	18.77 (3.05)				
C Surgery	1248 (31.6)	18.48 (2.67)				
D Gynecology and pediatrics	408 (10.3)	18.99 (1.86)				
E Others	208 (5.3)	18.69 (2.40)				
Organization workload			N/A	10.977 (39, 51)	<.001	C>B>A
A Excessive	84 (2.1)	18.12 (3.28)				
B Heavy	2194 (55.5)	19.24 (2.91)				
C Normal	1674 (42.4)	19.93 (2.58)				
Position			N/A	49.007 (39, 51)	<.001	D>C>A-B
A Nurse	2656 (67.2)	18.77 (2.61)				
B Specialist nurses	768 (19.4)	18.43 (1.36)				
C Head nurse	388 (9.8)	19.76 (3.45)				
D Director of nursing	140 (3.5)	20.14 (2.42)				
Professional titles			N/A	57.380 (39, 51)	<.001	C>B>A
A Primary	2164 (54.8)	18.73 (2.63)				
B Intermediate	800 (20.2)	19.10 (2.63)				
C Senior	988 (25)	19.86 (3.09)				
Years of working			N/A	16.178 (39, 51)	<.001	C>A-B D>A-B
A 1-5	1176 (29.8)	18.74 (2.78)				
B 5-10	776 (19.6)	18.86 (2.47)				
C 11-20	756 (19.1)	19.21 (3.05)				
D >20	1244 (31.5)	19.47 (2.78)				
Personal income (RMB^b^^c^ per month)			N/A	38.478 (39, 51)	<.001	B>A B>C
A 1-5000	2160 (54.7)	18.73 (2.60)				
B 5000-10,000	1516 (38.4)	19.69 (2.94)				
C >10,000	276 (6.9)	18.59 (3.02)				
Total	3952 (100)	18.87 (2.49)				

a N/A: not applicable.

b RMB: Renminbi (Chinese Yuan), the official currency of China.

c A currency exchange rate of RMB 1=US $0.71 is applicable.

**Table 5.** Perceived behavioral control to Internet Plus Nursing Service (IPNS) of primary health care nurses (N=3952).

		Perceived behavioral control scores				
Items	Participants, n (%)	Mean (SD)	*t* test *(df)*	*F* test *(df)*	*P* value	Post hoc
Gender			–25.773 (74.867)	N/A^a^	<.001	N/A
Female	3884 (98.2)	26.27 (3.58)				
Male	68 (1.8)	32.65 (1.98)				
Age (years)			N/A	6.171 (39, 51)	.002	B>A C>A
A <30	1676 (42.4)	25.26 (3.85)				
B 30‐40	972 (24.6)	26.74 (3.61)				
C >40	1304 (33)	26.27 (3.41)				
Education background			–1.984 (2167.088)	N/A	.047	N/A
Associate’s degree	1228 (31.1)	26.21 (3.91)				
Bachelor’s degree	2724 (68.9)	26.46 (3.53)				
Departments			N/A	46.408 (39, 51)	<.001	A>B-E
A Nursing management	564 (14.3)	27.6 (3.29)				
B Internal medicine	1452 (36.1)	25.94 (3.71)				
C Surgery	1248 (31.6)	25.71 (3.86)				
D Gynecology and pediatrics	408 (10.3)	25.43 (2.67)				
E Others	208 (5.3)	25.31 (3.49)				
Organization workload			N/A	22.801 (39, 51)	<.001	B>A C>A
A Excessive	84 (2.1)	24.87 (4.62)				
B Heavy	2194 (55.5)	26.53 (3.76)				
C Normal	1674 (42.4)	26.79 (3.41)				
Position			N/A	17.95 (39, 51)	<.001	D>A-C
A Nurse	2656 (67.2)	26.28 (3.59)				
B Specialist nurses	768 (19.4)	26.40 (2.53)				
C Head nurse	388 (9.8)	26.33 (4.25)				
D Director of nursing	140 (3.5)	27.57 (2.83)				
Professional titles			N/A	26.882 (39, 51)	<.001	C>B>A
A Primary	2164 (54.8)	25.50 (3.64)				
B Intermediate	800 (20.2)	26.38 (3.86)				
C Senior	988 (25)	26.78 (3.42)				
Years of working (years)			N/A	1.82 (39, 51)	.141	N/A
A 1-5	1176 (29.8)	26.54 (4.02)				
B 5-10	776 (19.6)	26.28 (3.30)				
C 11-20	756 (19.1)	26.48 (3.80)				
D >20	1244 (31.5)	26.23 (3.40)				
Personal income (RMB^b^^c^ per month)			N/A	1.484 (39, 51)	.227	N/A^a^
A 1-5000	2160 (54.7)	26.29 (3.69)				
B 5000-10,000	1516 (38.4)	26.48 (3.64)				
C >10,000	276 (6.9)	26.57 (3.50)				
Total	3952 (100)	25.67 (3.48)				

a N/A: not applicable

b RMB: Renminbi (Chinese Yuan), the official currency of China.

c A currency exchange rate of RMB 1=US $0.71 is applicable.

## Discussion

### Principal Findings and Comparison With Previous Works

European countries have been leading the way in implementing innovative practices in IPNS, showcasing various models that enhance patient care while tackling the challenges of an aging population and limited health care resources. For instance, the Netherlands and Sweden have successfully integrated digital health solutions into their health care systems, enabling remote monitoring and telehealth consultations that effectively manage chronic diseases. A meta-analysis revealed that residents in Western European regions, where digital health infrastructure is more advanced, showed significantly higher awareness and willingness to engage in IPNS [18]. These innovations not only improve access to care but also empower patients to take an active role in managing their health. Beyond remote consultations, European nations have leveraged mobile applications to facilitate communication between patients and health care providers, ensuring timely interventions and support. A notable example comes from Germany, where a study demonstrated the successful implementation of a web-based platform that enabled seamless communication between palliative care teams and patients, ultimately enhancing the quality of care provided [19]. Additionally, countries like Denmark have embraced a holistic approach to IPNS by integrating social care with health services, addressing the broader needs of patients and improving overall health outcomes. The effectiveness of these innovative practices is highlighted by the positive feedback from both patients and health care professionals. As these countries continue to refine their models, there is a growing emphasis on ensuring that all patients can benefit from these advancements in health care delivery [20].

This study is, to our knowledge, the first to assess the participation intentions of primary health care nurses in IPNS using a professional scale. Nurses in primary health institutions are crucial for the success of IPNS, as their attitudes toward participation directly influence the effectiveness of the program. In general, the study found that primary health care nurses exhibited a medium-high level of intention to participate in IPNS.

A majority of nurses showed a positive attitude toward participating in IPNS, viewing it as an opportunity for professional development and self-actualization. They believed that IPNS could enhance the value of nursing, improve their personal image, and increase their income. However, despite the positive outlook, more than half of the nurses expressed concerns that the risks associated with IPNS, such as medical risks, personal safety risks, and user information security risks, might outweigh its benefits. These findings are consistent with the results of previous studies, which highlighted concerns regarding the risks of internet-based health care services [21]. To address these concerns, it is crucial to improve relevant laws and regulations, enhance the monitoring of the service process, and establish robust safety protocols to ensure both the nurses’ and patients’ security. Strengthening emergency response mechanisms and clarifying legal protections will help mitigate perceived risks and improve nurses’ willingness to participate.

In terms of subjective norms, most of the nurses expressed a willingness to participate in IPNS based on the recommendations of hospital leaders, colleagues, and family members. The guidance from hospital leaders and experts emerged as a major factor shaping nurses’ willingness to participate, with most nurses acknowledging its substantial impact on their decision-making. This finding underscores the importance of leadership and organizational support in driving the adoption of new health care models like IPNS. Furthermore, a significant portion of nurses (n=1708; 43.2%) indicated that time constraints might hinder their participation. Time management challenges, including the time required for traveling and completing service-related tasks, could potentially reduce nurses’ engagement with IPNS [22]. To mitigate these issues, health care institutions and internet platforms should consider providing sufficient support to reduce time burdens. This could include flexible scheduling, allowing nurses to manage their work hours more efficiently, and utilizing big data to optimize care delivery logistics [23].

Regarding perceived behavioral control, the majority of nurses expressed positive perceptions of the policies, risks, and technology associated with IPNS, which indicates a favorable attitude toward this new model. However, more than half of the respondents were concerned that the change in the physical location of nursing activities (from the hospital to patients’ homes) could negatively impact the quality of care. This concern is understandable given the limited resources available in home settings, such as inadequate medical equipment and medications [24]. Moreover, the regulations in China currently stipulate that nurses should perform their duties at their registered workplace, which is inconsistent with the flexible practice model of IPNS. This regulatory mismatch raises concerns about potential “illegal medical practice” and could discourage nurses from participating in IPNS [25]. To address these concerns, there is a need for clear legal frameworks that support multisite practice, as well as regulations that ensure home environments meet the necessary standards for delivering safe and effective nursing care.

Despite the considerable conveniences brought by the widespread adoption of IPNS, this innovative care model has also raised significant safety concerns and implementation challenges. Nurses providing home-based care often encounter risks to their personal safety and face difficulties in safeguarding patient privacy. Research indicates that nursing professionals generally exhibit a high level of risk awareness regarding IPNS, with over 51% of frontline nurses recognizing moderate-to-high risk levels associated with this service [20]. Additionally, critical issues such as cybersecurity vulnerabilities of service platforms and inconsistent service quality standards have emerged. The current absence of comprehensive policies and uniform regulations has led to disparities in quality, which negatively affect patient trust and participation rates. These challenges highlight the urgent need to improve safety protections for nursing staff, establish robust service standards, and strengthen policy frameworks to ensure the sustainable development of IPNS. In response, several high-income countries have implemented institutional safeguards to ensure service quality and safety in digital nursing services. For example, Germany and Switzerland have created specific legislative and policy frameworks that clearly outline nurses’ responsibilities and safety standards in telenursing contexts, providing protections comparable to those in traditional care models [21]. Finland has successfully integrated information technology with nursing services through a centralized digital platform that facilitates remote patient monitoring and personalized care delivery [20]. Furthermore, many European nations have established dedicated regulatory bodies to oversee and evaluate the implementation of internet-based nursing services, ensuring compliance with national health care standards.

The study also revealed that male nurses were more likely to participate in IPNS than female nurses. From a socioeconomic standpoint, male nurses in China frequently encounter stronger traditional pressures to serve as the main family providers, potentially motivating them to pursue supplementary earnings through flexible work arrangements like IPNS [26]. This phenomenon is further compounded by deeply ingrained cultural stereotypes in Chinese society that traditionally link men with outdoor occupations while associating women with indoor caregiving roles, which may subtly shape their willingness to participate in such work opportunities. These intersecting factors of economic necessity and unconscious gender role expectations create a complex backdrop for understanding male nurses’ engagement with alternative income sources in China’s health care landscape. In contrast, female nurses may be more concerned about personal safety, particularly when providing care in patients’ homes, as this setting could expose them to potential risks. While male nurses generally perceived lower physical vulnerability. These gender differences suggest that targeted interventions may be needed to address the specific concerns of female nurses, such as implementing safety measures like one-click alarm systems to ensure nurses’ personal security during home visits [27].

Nurses with more than 11 years of experience and those over 30 years old showed a higher willingness to participate in IPNS. These nurses likely have greater confidence in their clinical skills and emergency response abilities, which makes them more comfortable with the challenges of providing care in home settings. This finding is consistent with previous research that suggested that experienced nurses tend to be more adaptable to new working environments [28]. Consequently, experienced nurses could play a central role in the expansion of IPNS, particularly in managing complex cases and addressing potential challenges that arise in home care situations.

Furthermore, nurses with higher educational qualifications (bachelor’s degree or above) were more likely to participate in IPNS. This could be attributed to their broader knowledge base and openness to adopting new technologies and models of care [29,30]. Nurses in nursing management roles also exhibited a higher intention to participate, likely due to their leadership positions and a deeper understanding of the potential benefits of IPNS for the health care system. The high level of interest in IPNS among nurses in management positions could also reflect their desire to improve nursing services in response to the growing demand for home health care.

In terms of organizational workload, nurses with lighter workloads were more likely to participate in IPNS. This finding aligns with previous studies that have shown that high workload pressures discourage participation in supplementary health care programs [13]. To encourage more nurses to engage in IPNS, health care institutions must balance the workload of primary health care nurses and ensure that their participation in IPNS does not lead to burnout or excessive strain. Offering training and incentive programs could also help increase nurses’ motivation to participate in this emerging model of care [31].

Finally, nurses with a personal income between 5000 and 10,000 RMB per month expressed the highest intention to participate in IPNS. This group of nurses, who are typically more established in their careers, may be more motivated to participate in IPNS as a means of supplementing their income and improving their financial stability. For nurses with higher incomes, the opportunity to participate in IPNS may be less appealing due to the additional workload it entails, while nurses with lower incomes may feel less confident in their ability to provide care in a new and potentially challenging environment.

### Limitations

This study has several limitations. First, the sample was limited to nurses from Jiangsu Province, which may affect the generalizability of the findings. Second, while we examined key sociodemographic factors, the study did not assess several important psychosocial and organizational determinants that may influence participation intentions, including job satisfaction, perceived meaningfulness of work, professional autonomy, competence beliefs, or perceived social impact of IPNS services—all of which have been shown to affect health care professionals’ engagement in innovative practices. Additionally, the study did not explore whether measured intentions translated into actual IPNS engagement, as this likely depends on contextual, organizational, and policy factors that require further investigation. Further research should employ longitudinal designs with more diverse national samples while incorporating comprehensive assessments of both structural and individual psychological factors to better understand IPNS participation dynamics.

### Conclusions

This study highlights the medium-high level of participation intention among primary health care nurses in IPNS and identifies several demographic and work-related factors that influence their willingness to participate. These findings underscore the importance of addressing concerns related to time, safety, and the regulatory framework to increase nurse engagement in IPNS.

Policymakers should adopt a comprehensive strategy to improve IPNS participation by implementing strong safety measures such as real-time GPS tracking and emergency alerts, along with clear legal guidelines to define liability and safeguard nurses during home visits. To enhance efficiency and reduce burnout, artificial intelligence–powered scheduling tools should be used to intelligently distribute workloads based on travel distance, case difficulty, and nurse expertise. Additionally, a tiered incentive program should be introduced, offering middle-income nurses financial rewards like bonuses or tax breaks while providing experienced nurses with career advancement opportunities and leadership roles in IPNS initiatives.

These efforts should be supported by continuous nurse feedback channels and public education campaigns to ensure long-term success, fostering a system that tackles safety risks, workload challenges, and financial concerns while ensuring fair service access in both urban and rural communities. This well-rounded approach will not only increase nurse involvement but also elevate the overall quality and availability of IPNS for patients.

Policymakers and health care institutions should focus on creating supportive environments, providing adequate training, and offering incentives to encourage more nurses to participate in this promising model of home health care.

## Abbreviations

IPNS: Internet Plus Nursing Service
